# Exponential Growth Bias of Infectious Diseases: Protocol for a Systematic Review

**DOI:** 10.2196/37441

**Published:** 2022-10-24

**Authors:** Siv Hilde Berg, Daniel Adrian Lungu, Kolbjørn Brønnick, Stig Harthug, Jo Røislien

**Affiliations:** 1 Centre for Resilience in Healthcare Faculty of Health Sciences University of Stavanger Stavanger Norway; 2 Department of Research and Development Huakeland University Hospital Bergen Norway

**Keywords:** exponential growth bias, pandemic, infectious diseases, COVID-19, health communication, risk communication

## Abstract

**Background:**

Humans struggle to grasp the extent of exponential growth, which is essential to comprehend the spread of an infectious disease. Exponential growth bias is the tendency to linearize exponential functions when assessing them intuitively. Effective public health communication about the nonlinear nature of infectious diseases has strong implications for the public’s compliance with strict restrictions. However, there is a lack of synthesized knowledge on the communication of the exponential growth of infectious diseases and on the outcomes of exponential growth bias.

**Objective:**

This systematic review identifies, evaluates, and synthesizes the findings of empirical studies on exponential growth bias of infectious diseases.

**Methods:**

A systematic review will be conducted using the PRISMA-P (Preferred Reporting Items for Systematic Review and Meta-Analysis Protocols) 2015 statement. Eligibility criteria include empirical studies of exponential growth bias of infectious diseases regardless of methodology. We include studies both with and without interventions/strategies. For information sources, we include the following five bibliographic databases: MEDLINE, Embase, Cochrane Library, PsychINFO, and Web of Science Core Collection. The risk of bias will be assessed using RoB 2 (Risk of Bias 2) and STROBE (Strengthening the Reporting of Observational Studies in Epidemiology). Data synthesis will be achieved through a narrative synthesis.

**Results:**

By February 2022, we included 11 experimental studies and 1 cross-sectional survey study. Preliminary themes identified are the presence of exponential growth bias, the effect of exponential growth bias, and communication strategies to mitigate exponential growth bias. Data extraction, narrative synthesis, and the risk of bias assessment are to be completed by February 2023.

**Conclusions:**

We anticipate that this systematic review will draw some lines related to how people comprehend and misperceive exponential growth and its consequences for infectious disease mitigation and communication. Furthermore, the study will conclude with the limitations of the research and suggestions for future research.

**International Registered Report Identifier (IRRID):**

DERR1-10.2196/37441

## Introduction

The COVID-19 pandemic has unfortunately demonstrated the power of exponential growth and the need to understand why humans struggle to grasp the extent of a spreading infectious disease. Exponential growth bias is defined by Stango and Zinman [1] as “the pervasive tendency to linearize exponential functions when assessing them intuitively.” Exponential growth bias is a well-documented phenomenon that dates to the 1970s [2-4].

Exponential growth bias has been documented in numerous contexts and populations, using both experimental and observational methods [1]. Nonexperts underestimate exponential growth and trends, assuming that the growth is linear [2-4]. At the same time, many nonexperts overestimate their ability to estimate exponential growth [5]. The bias may seem robust, as the misconception of exponential growth is occurring even among people with an advanced education in mathematics [6,7]. Even heads of state either failed to respond or downplayed the spread of the virus in the early phases of the COVID-19 pandemic [8].

Prior epidemiological studies have documented how the spread of infectious diseases, especially in the initial stages, often follows an exponential function [9-11]. Early in the COVID-19 pandemic, Li et al [9] documented how the spread of COVID-19 was exponential. In West Africa, outbreaks of Ebola showed near-exponential growth in the districts of Margibi in Liberia and Bombali and Bo in Sierra Leone [12].

Effective public health communication of the nonlinear nature of infectious diseases has strong implications for public compliance with restrictions. However, there is little synthesized knowledge on the communication of exponential growth of infectious diseases and the outcomes of the exponential growth bias. This systematic review identifies, evaluates, and synthesizes the findings of empirical studies on the exponential growth bias of infectious diseases. The review questions are:

- What are the consequences of exponential growth bias of infectious diseases?
- What strategies can mitigate exponential growth bias of infectious diseases?

## Methods

A systematic review will be conducted using the PRISMA-P (Preferred Reporting Items for Systematic Review and Meta-Analysis Protocols) 2015 statement [13].

### Eligibility Criteria

Consistent with the aim to identify findings of empirical studies, we include empirical studies regardless of applied methodology (eg, randomized controlled trials, nonrandomized studies [quasi-experimental trials], survey studies, and qualitative studies). Commentaries, reviews, opinion pieces, or other papers not reporting primary empirical research are excluded. Only English-language peer-reviewed studies are included. PICO (Problem, Intervention or Exposure, Comparison, Outcome) is used to define our rationale and eligibility criteria.

#### Problem

We include studies of exponential growth bias of infectious diseases. We define exponential growth bias as “the pervasive tendency to linearize exponential functions when assessing them intuitively” [1]. We exclude studies of exponential growth of infectious diseases that did not study the human perception of exponential growth (eg, statistical, prediction, or selection bias). Likewise, studies of cognitive biases not related to exponential growth are excluded (eg, anchoring bias). Infectious diseases include outbreaks, epidemics, and pandemics in real-life or fictional cases. Studies of exponential growth bias in other contexts (eg, economy) are excluded.

#### Intervention or Exposure

We included studies both with and without interventions​/​strategies to mitigate exponential growth bias.

#### Outcome

The studies have to report on the presence of exponential growth bias of infectious diseases to be included. Additional outcomes of interest, which are not necessary to be eligible for inclusion, are the outcomes of strategies to mitigate exponential growth bias of infectious diseases and the outcomes of exponential growth bias of infectious diseases.

### Search Strategy and Information Sources

A presearch provided a limited number of hits. To increase the sensitivity of the search, we include five bibliographic databases: MEDLINE, Embase, Cochrane Library, PsychINFO, and Web of Science Core Collection. Based on initial literature searches, several papers were selected for further cited reference searches in Web of Science as a supplement to the traditional searches. In line with the PRISMA (Preferred Reporting Items for Systematic Reviews and Meta-Analyses) guidelines [13], the selection of databases, search terms, and search methodology was determined in collaboration with a university library technician (Geir Strandenæs Larsen) who designed the final search. Author SH gave feedback on the search terms related to infectious diseases. The final search results were exported to EndNote, and Geir Strandenæs Larsen removed the duplicates. The main database search was conducted on January 5 and 7, 2022. We searched using the terms *exponential growth bias*, *exponential growth prediction bias forecast​/​misconception​/​misperception of exponential growth*, AND *infectious diseases*, epidemic*, pandemic*, outbreak*, or contagious​/​transmissible​/​communicable disease*. No filters or limits were added in our literature searches (eg, language, peer-review, or publication date range). No searches of gray literature were conducted. The full electronic search strategy for all databases is shown in Multimedia Appendix 1.

### Selection of Sources of Evidence

As of February 2022, the search yielded 585 results. After removing duplicates, there were 365 unique results. The full text of 50 articles were read and assessed for eligibility; 39 were excluded, and 11 were included in the review.

One reviewer (SHB) undertook the screening and inclusion, in dialogue with author JR. All records were added to Rayyan (software for intelligent systematic review). SHB assessed abstracts and full-text articles using the eligibility criteria.

### Data-Charting Process

Data will be extracted by one researcher (SHB) and will be checked by a second researcher (DAL or JR). Data from included papers will be extracted to a matrix prior to synthesis: author, year of publication, aim, sample size, origin, methods, and results. Pilot-testing of the data extraction form has been conducted by extracting information from 3 studies. The extracted data will be displayed in a table, and the content of the table will be validated by JR and DAL.

### Synthesis Methods

Due to the heterogeneity of the studies regarding methodology and outcome measures, a statistical meta-analysis was considered inappropriate [14]. A narrative synthesis is used when statistical meta-analysis is not feasible and refers to an approach for systematic reviews and synthesis of findings from multiple studies that relies primarily on the use of words and text to summarize and explain the findings of the synthesis [15]. When used in a systematic review, a narrative synthesis focuses on a wide range of questions, not only those relating to the effectiveness of a specific intervention [15]. Data synthesis will be achieved through a narrative synthesis using the four stages of data synthesis proposed by Whittemore and Knafl [16]. The results relevant to the review question will be summarized, coded inductively, and initially categorized in accordance with the review questions (first stage). Data displays of the categories will be made to visualize patterns and relationships among data (second stage). The themes will be verified by keeping track of the primary source data (fourth stage). The analysis will be conducted by SHB. JR and DAL will read the articles and validate the analysis.

### Quality Appraisal

Risk of bias will be assessed using the ROBINS-I (Risk of Bias in Nonrandomized Studies of Interventions), the preferred tool to be used in Cochrane reviews for nonrandomized studies of interventions [17]. The randomized controlled trials will be assessed using Cochrane Collaboration’s RoB 2 (Risk of Bias 2) tool for randomized trials [18]. The randomized and nonrandomized studies will be assessed as critical, serious, moderate, low, or no information (templates shown in Tables 1 and 2). The STROBE (Strengthening the Reporting of Observational Studies in Epidemiology) statement checklist will be used for quality assessment of observational (cohort and cross-sectional) studies [19]. The risk of bias assessment will be conducted by authors SHB, JR, DAL, and KB.

**Table 1:** Template for the risk of bias assessment in accordance to ROBINS-I (Risk of Bias in Nonrandomized Studies of Interventions) [17].

	Studies
Confounding	—^a^
Selection of participants	—
Classifications of interventions	—
Deviations from interventions	—
Missing data	—
Measurement of outcome	—
Selection of reported results	—
Overall risk of bias	—

^a^Reference numbers will be included here.

**Table 2:** Template for risk of bias assessment in accordance to RoB 2 (Risk of Bias 2) [18].

	Studies
Randomization process	—^a^
Deviations from interventions	—
Missing data	—
Measurement of outcome	—
Selection of reported results	—
Overall risk of bias	—

^a^Reference numbers will be included here.

## Results

**Figure 1:**
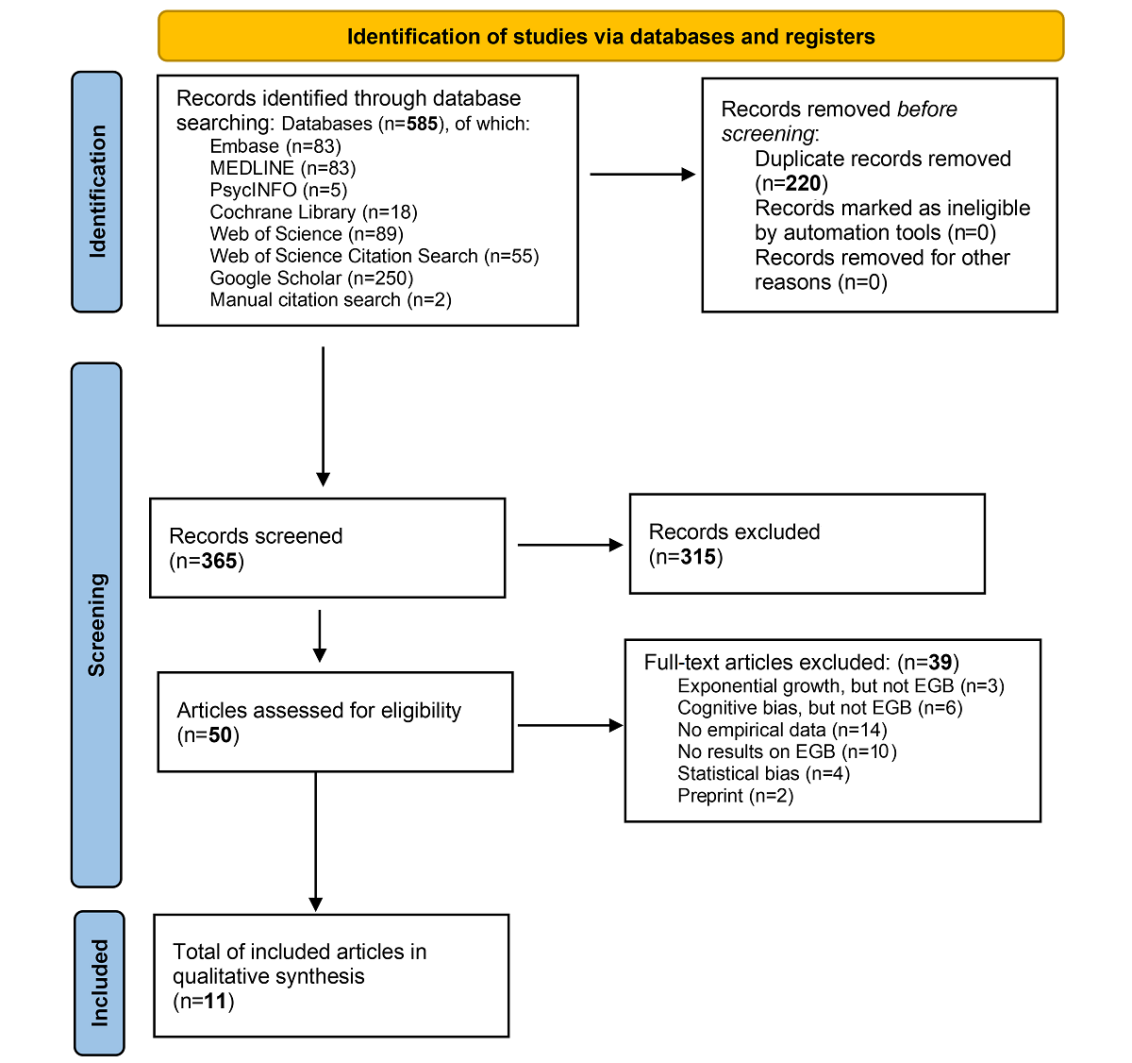
PRISMA (Preferred Reporting Items for Systematic Reviews and Meta-Analyses) flow diagram for studies included in the present review of exponential growth bias (EGB).

As of February 2022, we have included 11 studies (see the PRISMA flow diagram in Figure 1). This comprises 11 experimental studies and 1 cross-sectional survey study. The preliminary themes identified are the presence of exponential growth bias, the effect of exponential growth bias, and communication strategies to mitigate exponential growth bias. Data extraction, narrative synthesis, and the risk of bias assessment is expected to be completed by February 2023.

## Discussion

This review presents an unbiased summary and analysis of the evidence of the exponential growth bias of infectious diseases. We believe that the review will provide useful information to guide future research and public health communication strategies. The anticipated main findings of this study will document the presence of exponential growth bias of infectious diseases and its personal and societal consequences, and identify communication strategies that may mitigate the exponential growth bias of infectious diseases. However, since this research is in its early development, we expect to find few methodologically diverse studies. Although we cannot conduct a meta-analysis and statistical synthesis of the outcomes in this systematic overview, we expect this review to generate scholarly discussion and research. Thus, the discussion will focus on the limitations of the research and suggestions for future research in the fields of health communication, media studies, psychology, and mathematics. The review is expected to be submitted to the *Journal of Medical Internet Research* in June 2023.

## Appendix

Multimedia Appendix 1 Search strategy.

## Abbreviations

PICO: Problem, Intervention or Exposure, Comparison, Outcome
PRISMA: Preferred Reporting Items for Systematic Reviews and Meta-Analyses
PRISMA-P: Preferred Reporting Items for Systematic Review and Meta-Analysis Protocols
RoB 2: Risk of Bias 2
ROBINS-I: Risk of Bias in Nonrandomized Studies of Interventions
STROBE: Strengthening the Reporting of Observational Studies in Epidemiology
