# Supplementary material for: Exponential Growth Bias of Infectious Diseases: Protocol for a Systematic Review
Source: JMIR Res Protoc. 2022 Oct 24;11(10):e37441. doi: 10.2196/37441 (PMC9641520; doi:10.2196/37441)
Supplement: Multimedia Appendix 1 [file resprot_v11i10e37441_app1.docx]

**Additional file 1**

| **Database / Source** | Embase <1974 to 2022 January 05> |
| --- | --- |
| **Search Date** | 06.01.2022 |
| **Search History** | 1 ((exponential-growth* or exponential* or growth* or prediction* or forecast*) adj3 (bias* or misconception* or misconseption* or misperception* or misperseption*)).mp. 1445  2 ((exponential adj2 growth*) and (bias* or misconception* or misconseption* or misperception* or misperseption*)).mp. 113  3 1 or 2 1543  4 pandemic/ or exp epidemic/ or communicable disease/ 240753  5 (((contagious or transmissible or communicable) adj3 (disease* or ill* or sick*)) or pandemic* or epidemic* or infect* or outbreak*).mp. 3348021  6 4 or 5 3348106  7 3 and 6 83 |
| **Number of Results** | 83 (before removal of duplicates) |
| **Comment** |  |

| **Database / Source** | Ovid MEDLINE(R) ALL <1946 to January 05, 2022> |
| --- | --- |
| **Search Date** | 06.01.2022 |
| **Search History** | 1 ((exponential-growth* or exponential* or growth* or prediction* or forecast*) adj3 (bias* or misconception* or misconseption* or misperception* or misperseption*)).mp. 1329  2 ((exponential adj2 growth*) and (bias* or misconception* or misconseption* or misperception* or misperseption*)).mp. 108  3 1 or 2 1423  4 Pandemics/ or Epidemics/ or exp Communicable Diseases/ 596779  5 (((contagious or transmissible or communicable) adj3 (disease* or ill* or sick*)) or pandemic* or epidemic* or infect* or outbreak*).mp. 2728612  6 4 or 5 2852377  7 3 and 6 83 |
| **Number of Results** | 83 (before removal of duplicates) |
| **Comment** |  |

| **Database / Source** | APA PsycInfo <1806 to December Week 4 2021> |
| --- | --- |
| **Search Date** | 06.01.2022 |
| **Search History** | 1 ((exponential-growth* or exponential* or growth* or prediction* or forecast*) adj3 (bias* or misconception* or misconseption* or misperception* or misperseption*)).mp. 576  2 ((exponential adj2 growth*) and (bias* or misconception* or misconseption* or misperception* or misperseption*)).mp. 29  3 1 or 2 587  4 pandemics/ or epidemics/ or exp infectious disorders/ 76791  5 (((contagious or transmissible or communicable) adj3 (disease* or ill* or sick*)) or pandemic* or epidemic* or infect* or outbreak*).mp. 90475  6 4 or 5 114191  7 3 and 6 5 |
| **Number of Results** | 5 (before removal of duplicates) |
| **Comment** |  |

| **Database / Source** | Cochrane Library |
| --- | --- |
| **Search Date** | 06.01.2022 |
| **Search History** | #1 ((exponential NEXT growth* or exponential* or growth* or prediction* or forecast*) NEAR/3 (bias* or misconception* or misconseption* or misperception* or misperseption*)) 58  #2 ((exponential NEAR/2 growth*) and (bias* or misconception* or misconseption* or misperception* or misperseption*)) 9  #3 #1 or #2 67  #4 MeSH descriptor: [Pandemics] explode all trees 366  #5 MeSH descriptor: [Epidemics] explode all trees 407  #6 MeSH descriptor: [Communicable Diseases] explode all trees 16577  #7 (((contagious or transmissible or communicable) NEAR/3 (disease* or ill* or sick*)) or pandemic* or epidemic* or infect* or outbreak*) 161443  #8 #4 or #5 or #6 or #7 164503  #9 #3 and #8 18 |
| **Number of Results** | 18 (before removal of duplicates) |
| **Comment** |  |

| **Database / Source** | Web of Science Core Collection Editions = A&HCI , ESCI , SCI-EXPANDED , SSCI |
| --- | --- |
| **Search Date** | 07.01.2022 |
| **Search History** | 5 #3 AND #4  4 TS=(((contagious or transmissible or communicable) NEAR/2 (disease* or ill* or sick*)) or pandemic* or epidemic* or infect* or outbreak*)  3 #1 OR #2  2 TS=((exponential NEAR/1 growth*) and (bias* or misconception* or misconseption* or misperception* or misperseption*))  1 TS=((exponential-growth* or exponential* or growth* or prediction* or forecast*) NEAR/2 (bias* or misconception* or misconseption* or misperception* or misperseption*)) |
| **Number of Results** | 89 (before removal of duplicates) |
| **Comment** |  |

| **Database / Source** | Web of Science Core Collection – Citation Search Editions = A&HCI , ESCI , SCI-EXPANDED , SSCI |
| --- | --- |
| **Search Date** | 07.01.2022 |
| **Search History** | Based on initial scoping literature searches a selection of relevant papers were made for further Cited Reference searches in Web of Science as a supplement to the systematic searches using traditional search strategies. Of the selected relevant papers, the papers below (4 in total) were papers with more than 1 citing article in Web of Science. All citing articles (55 in total) for the following papers were included for screening in this study:  *Lammers, J., Crusius, J., & Gast, A. (2020). Correcting misperceptions of exponential coronavirus growth increases support for social distancing. Proceedings of the National Academy of Sciences of the United States of America, 117(28), 16264-16266. https://doi.org/http://dx.doi.org/10.1073/pnas.2006048117*  *Banerjee, R., Bhattacharya, J., & Majumdar, P. (2021). Exponential-growth prediction bias and compliance with safety measures related to COVID-19. Social Science & Medicine, 268, 9, Article 113473.* [*https://doi.org/10.1016/j.socscimed.2020.113473*](https://doi.org/10.1016/j.socscimed.2020.113473)  *Schonger, M., & Sele, D. (2020). How to better communicate the exponential growth of infectious diseases. PLoS ONE, 15(12), 13, Article e0242839. https://doi.org/10.1371/journal.pone.0242839*  *Romano, A., Sotis, C., Dominioni, G., & Guidi, S. (2020). The scale of COVID‐19 graphs affekts understanding, attitudes, and policy preferences. Health economics, 29(11), 1482-1494* |
| **Number of Results** | 55 (before removal of duplicates) |
| **Comment** |  |

| **Database / Source** | Google Scholar |
| --- | --- |
| **Search Date** | 07.01.2022 |
| **Search History** | Search 1: exponential growth bias pandemic  Search 2: exponential growth bias epidemic  Search 3: exponential growth bias infectious diseases  Search 4: exponential growth bias outbreak |
| **Number of Results** | 200 (before removal of duplicates) |
| **Comment** | Several searches using short search query strings (4 in total) were carried out in Google Scholar. This is because Google Scholar tends to rank results sub optimally if longer search query strings are used. As Google Scholar uses Google Page Rank to sort references by relevance, the top 50 ranked articles were selected for each search, resulting in a total of 200 references before removal of duplicates. |

| **Database / Source** | Google Scholar |
| --- | --- |
| **Search Date** | 29.11.2021 |
| **Search History** | Search 5: exponential growth bias |
| **Number of Results** | 50 (before removal of duplicates) 1 after removing duplicate |
| **Comment** | Manual screening |

| **Database / Source** | Google Scholar |
| --- | --- |
| **Search Date** | 29.11.2021 |
| **Search History** | Citations of: Exponential-growth prediction bias and compliance with safety measures related to COVID-19 |
| **Number of Results** | 1 (before removal of duplicates) |
| **Comment** | Manual screening  One Article included, missed in web of science citation screening. Romano et al |

| **Database / Source** | References |
| --- | --- |
| **Search Date** | 29.11.2021 |
| **Search History** | References of: Exponential-growth prediction bias and compliance with safety measures related to COVID-19 |
| **Number of Results** | 1 (before removal of duplicates) |
| **Comment** | Manual screening  One Article included from references . Fetzer et al |
